# Rapid evolution of phenotypic plasticity in patchy habitats

**DOI:** 10.1038/s41598-023-45912-8

**Published:** 2023-11-06

**Authors:** Nawsheen T. Promy, Mitchell Newberry, Davorka Gulisija

**Affiliations:** 1 Department of Computer Science, University of New Mexico, Albuquerque, USA; 2 Center for the Study of Complex Systems, University of Michigan, Ann Arbor, USA; 3 Department of Biology, University of New Mexico, 219 Yale Boulevard NE, 3566 Castetter Hall, Albuquerque, NM 87131 USA

**Keywords:** Computational biology and bioinformatics, Evolution

## Abstract

Phenotypic plasticity may evolve rapidly, enabling a population’s persistence in the face of sudden environmental change. Rapid evolution can occur when there is considerable genetic polymorphism at selected loci. We propose that balancing selection could be one of the mechanisms that sustain such polymorphism for plasticity. We use stochastic Monte Carlo simulations and deterministic analysis to investigate the evolution of a plasticity modifier locus in structured populations inhabiting favorable and adverse environments, i.e. patchy habitats. We survey a wide range of parameters including selective pressures on a target (structural) locus, plasticity effects, population sizes, and migration patterns between demes including periodic or continuous bidirectional and source-sink dynamics. We find that polymorphism in phenotypic plasticity can be maintained under a wide range of environmental scenarios in both favorable and adverse environments due to the balancing effect of population structure in patchy habitats. This effect offers a new plausible explanation for the rapid evolution of plasticity in nature: Phenotypic plasticity may rapidly evolve from genetic variation maintained by balancing selection if the population has experienced immigration from populations under different selection regimes.

## Introduction

Phenotypic plasticity is the ability of a single genotype to produce different phenotypes in response to different environmental conditions^1–3^. Plasticity is known to appear in adverse habitats where it buffers the fitness-reducing impacts of selection on maladaptive phenotypes^2,4–6^ and is considered one of the key abilities that allow populations to persist in the face of drastic environmental change or establish in new habitats before constitutive adaptation at a structural locus can occur^5,7,8^. For example, invasive populations that successfully colonize new habitats have been observed to have higher levels of plasticity in fundamental fitness components than non-invaders^9^. These features of plasticity make it a primary candidate for enabling populations to survive accelerated climate change, range shift of competitors, habitat expansions, or other sudden shifts in the environment. Yet despite its fundamental importance in sustaining biodiversity in the changing world, much is still unclear about how plasticity arises in nature.

Plasticity may have a genetic basis and, therefore, evolve^1^. Two genetic mechanisms of plastic response have been widely accepted and supported empirically: environmentally sensitive loci and epistatically interacting loci^10^. By the former mechanism for example, the environment might induce epigenetic modifications near a directly-selected structural coding sequence which results in plastic phenotypes^11,12^. The latter mechanism involves the interaction of loci, such as regulatory loci that affect target expression^10^, and hence constitutes a kind of epistasis. Theoretical studies have explored this type of epistasis using modifier locus models^6,13,14^. In such models, an allele at the modifier locus, such as a transcription factor or an epigenetic modifier, may affect the expression of a target locus so as to improve the phenotype or fitness in a particular environment. This improvement in turn makes the modifier allele selectively advantageous. The modifier allele remains selectively advantageous until the target locus evolves an optimal phenotype through means independent of environmental cues, such as through beneficial mutation and positive selection, i.e. constitutive adaptation. For example, in constant conditions, there is no longer any advantage to plasticity once constitutive adaptation occurs^5,15–17^. This means that to provide evolutionary rescue, plasticity must evolve more quickly^5^ than de novo constitutive beneficial mutations.

How phenotypic plasticity could evolve rapidly is unclear. Genetic variation provides one possible explanation because newly adaptive alleles would already be present in a population at the time of environmental change^18^. Furthermore, absolute change in allele frequency is greater if the initial allele frequency is already appreciable, due to the multiplicative nature of allele frequency change^19^. The speed at which both plastic and non-plastic responses can evolve in nature^9^ could therefore, at least in some conditions, be explained by genetic variation underlying the trait.

The necessary genetic variance may exist due to an abundance of mutations at mutation-selection balance if plasticity is a polygenic trait coded by numerous small-effect loci^5,20^. In that case plasticity could evolve rapidly due to minute changes at many loci in unison. However, mapping studies indicate that some variation in plasticity comes from a few loci of major effect^21–24^. These few loci collectively have a much smaller chance of mutating, while major effect alleles are more likely to be purged or fixed by directional selection. Thus, the mechanisms that maintain diversity at single or a few major effect loci are different from those that maintain diversity across the genome. Molecular evolution studies of single loci offer answers for how variation can be maintained at a gene level^13,25–27^.

Plasticity modifiers, however, might evolve differently from single directly-selected loci due to the distinct way in which plasticity affects fitness. Fitness effects of gene regulation by a modifier are indirect and dependent on both the environment and the target locus^13,14^. Plasticity may further come with fitness costs^5,15–17,28–30^. Fitness costs of plasticity may be associated for example with creating or maintaining the systems and structures necessary for osmo- or thermo-regulation^31^, sensing the environment, or maintaining genes and enzymes involved in modification. Plasticity may be selected against if the cost outweighs the benefit, such as when adverse conditions pass or when less-costly constitutive adaptation takes place at the target locus^13^. Even in the absence of direct costs, plasticity becomes neutral at best once alleles for constitutive adaptation are available.

Balancing selection is one key mechanism of maintaining elevated genetic variation that would enable rapid evolution. While conditions for balanced polymorphism are well known, the most obvious mechanisms, such as direct negative frequency-dependent selection^32^ or overdominance^33^, do not immediately fit the mode of selection on modifiers of phenotypic plasticity. Spatially and temporally varying environments, however, are ubiquitous in nature and have been shown to create opportunities for balancing selection^25–27,34^. Balancing selection for plasticity has been shown in temporally varying environments due to a genomic storage effect^13^. This genomic storage effect is a consequence of balanced polymorphism at the target locus, which generates heterogeneous genetic backgrounds required for the storage effect to apply to plasticity alleles. Plasticity in spatially varying environments has also been investigated. Classical models found that continuous reaction norms and developmental switches may be favored in metapopulations composed of multiple habitats^35^ and that some mixture of ancestral and stress-tolerant forms is an evolutionary stable state when stress-tolerant forms are able to invade a new habitat^36^. Models of the reaction norms themselves have shown that some combination of plastic and constitutive loci tend to underly multi-locus traits when both are allowed to evolve^37^, while other theoretical studies investigate constraints on plasticity^38,39^ or conditions under which plasticity is favored^40^. These studies nonetheless have not explicitly investigated conditions for balanced polymorphism under spatial heterogeneity.

Here, we investigate how spatial heterogeneity might induce balancing selection at a plasticity modifier locus in structured populations, and in turn promote rapid evolution of phenotypic plasticity. We develop a haploid, two locus, two allele, two deme population genetic model in which a modifier locus conveys a plastic response at a target (structural) locus underlying a trait under heterogeneous selection between the two habitat patches. This two-habitat model describes many natural situations in which selection might differ due to environmental change in one or another habitat. Such situations arise in naturally patchy habitats or when part of a population finds itself in an adverse habitat patch. Subpopulations that are not yet adapted to a local habitat can occur due to passive dispersal in plants^41^ or protists^42^, or due to anthropogenic disturbance^43,44^ such as global transport^45,46^ of large groups of invasive individuals in ballast water^47^. We model the effects of genotype and environment on fitness directly by specifying the fitness of each genotype in each environment, without explicitly modeling the phenotype. By assuming the modeled loci evolve independently of other loci in the genome^48^, this approach makes relatively weak assumptions about genetic architecture^49^.

To demonstrate balancing selection at the plasticity modifier locus, we use two lines of evidence. First, we derive an analytic solution for a stable polymorphic equilibrium at the plasticity locus in a deterministic model in terms of selection, plasticity effects, and migration rates between demes. Second, we observe whether stable long-term diversity at the modifier locus arises from single-mutant introductions in finite populations in forward-in-time stochastic simulations across a range of selective pressures, costs and benefits to plasticity, rates of periodic and continuous, one- and bi-directional migration between demes, and even and uneven population sizes.

We find balanced polymorphism at the plasticity modifier locus under a wide range of conditions provided that migration is present but limited, selection has opposite direction in each deme, and that neither costs nor benefits to plasticity are overwhelming such that the plasticity allele fixes or is lost. In contrast to previous results on temporally varying selection^13^, balanced polymorphism occurs when the target locus is monomorphic. Here, heterogeneous fitness effects at the target locus in different environments play the role of different genetic backgrounds underlying the genomic storage effect^13^. Balanced polymorphism at modifier loci in turn enables rapid evolution of plasticity by selection on standing variation following a sudden environmental change in a population. These results, therefore, suggest that balancing selection on plasticity modifiers due to migration between patchy habitats may offer a new explanation for many natural cases of rapid plasticity evolution in the face of sudden environmental change.

## Model and methods

### The finite population genetic model

We examine evolution at a plasticity modifier locus under a wide range of evolutionary scenarios in a haploid Wright-Fisher population. We simulate forward-in-time discrete-generation evolution with each generation consisting of migration, selection, recombination, and reproduction. Our main interest is the maintenance of variation in plasticity by selection and migration between demes rather than variation maintained by influx of new mutations. We therefore model what happens following a single-mutant introduction. These results are then easily extrapolated to the case of recurrent mutation. We model one mutation event at each of the plasticity and target loci per simulation run. We allow mutation at both loci at the same rate to account for interference between plastic and constitutive adaptation. Our analysis focuses on polymorphism at the plasticity locus, which determines its potential for rapid adaption.

At the beginning of each simulation run (generation *t* = 0), a population splits into two demes (subpopulations), or there is a sudden change of environments in one portion of the habitat, such that one deme now inhabits the ancestral favorable habitat while the other deme inhabits a derived adverse habitat patch. We assume that prior to this, a population was at its fitness optimum and therefore that both demes are monomorphic for the non-plastic allele (*m*) at the plasticity modifier locus and the ancestral allele (*a*) at the target locus. That is, adaptation to the new environment has not yet occurred. Then, a single novel mutant allele arises at random at one of the two loci in one of the two demes replacing an ancestral (*a*) allele with a derived allele (*d*) at a target locus or a non-plasticity allele (*m*) with a plasticity allele (*M*) at the modifier locus. The remaining monomorphic locus mutates in a random individual with a probability $N\mu$ in each generation until mutation at the remaining locus occurs once in a simulation run. This allows us to account for potential interference between adaptive dynamics at the two loci while allowing mutation at the two loci to arise independently. Note, we do not model recurrent mutation since we are concerned with the maintenance of variation following single mutant introductions. We then analyze and report whether this initial mutation event results in a stable polymorphic equilibrium, without recurrent mutation as in other theoretical work on balanced polymorphism^34^, although we later show results for a case with recurrent mutation as well.

In each generation, the frequency of the eight possible haplotypes in the meta-population *x*_*am1,*_* x*_*aM1,*_* x*_*dm1,*_* x*_*dM1,*_* x*_*am2,*_* x*_*aM2,*_* x*_*dm2,*_ and *x*_*dM2,*_ evolve under evolutionary forces of constant or periodic migration between the demes, spatially heterogenous selection, recombination between the two loci, and effects of genetic drift (reproduction) in the two demes. The list and description of the main variables used in the study are given in Table 1.

**Table 1:** Major mathematical symbols used in the study.

Symbol	Description
*N*_*i*_	The size of deme *i* (*i* = 1 or 2)
*e*_*i*_	Number of migrants leaving from deme *i* each *I* generations, i.e. per migration event
*s*_*i*_	Directional selection coefficient of a target allele in deme *i*
*p*_*hi*_	The plasticity effect—a net effect of the benefit and cost of the *M* allele at the plasticity modifier locus on the fitness of a target allele *h* in a deme *i*
*I*	A number of generations between periodic migration events

Migration allows *e*_1_ random individuals to migrate from the ancestral deme (*i* = 1) to the derived deme (*i* = 2) and *e*_2_ to migrate in the opposite direction, with the migration being either reciprocal when *e* = *e*_2_ = *e*_1_ or source-sink dynamics^50–52^ when *e*_2_ = 0. Migration occurs at each *I*th generation with no migration in between. Therefore *I* = 1 represents continuous migration and *I* > 1 represents periodic migration. We opted for a wide range of migration settings to accommodate a wide range of scenarios as could arise such as regularly mixing naturally fragmented habitats and irregular mass transports between distinct habitats due to global trade^47^.

We model an adapted-maladapted dynamic with opposite selection on the *a* and *d* alleles in the ancestral and derived demes. The haplotype frequencies at the beginning of generation *t* within each deme *i* (*i* = 1 or 2), are modified by selection so that the expected post-selection haplotype frequencies in generation *t* are $x_{{\text{ami,t}}}^{\prime } = x_{{\text{ami,t}}} \frac{{w_{{{\text{ami}}}} }}{{\overline{w}_{{i{\text{,t}}}} }}, x_{{\text{aMi,t}}}^{\prime } = x_{{\text{aMi,t}}} \frac{{w_{{{\text{aMi}}}} }}{{\overline{w}_{{i{\text{,t}}}} }}, x_{{\text{dmi,t}}}^{\prime } = x_{{\text{dmi,t}}} \frac{{w_{{{\text{dmi}}}} }}{{\overline{w}_{{i{\text{,t}}}} }},\;{\text{and}}\;x_{{\text{dMi,t}}}^{\prime } = x_{{\text{dMi,t}}} \frac{{w_{{{\text{dMi}}}} }}{{\overline{w}_{{i{\text{,t}}}} }}$.

Where

1 $$w_{{{\text{ami}}}} = 1 + s_{i} ,$$

2 $$w_{{{\text{aMi}}}} = 1 + s_{i} \left( {1 - p_{ai} } \right),$$

3 $$w_{{{\text{dmi}}}} = 1 - s_{i} ,$$

4 $$w_{{{\text{dMi}}}} = 1 - s_{i} \left( {1 - p_{di} } \right),$$

and

5 $$\overline{w}_{{i{\text{,t}}}} = x_{{\text{ami,t}}} w_{{{\text{ami}}}} + x_{{\text{aMi,t}}} w_{{{\text{aMi}}}} + x_{{\text{dmi,t}}} w_{{{\text{dmi}}}} + x_{{\text{dMi,t}}} w_{{\text{dMi }}} ,$$

where $s_{i}$ is the selection coefficient in the deme *i*, and is initially set to $s_{1} = s$ and $s_{2} = - s$. We later relax this assumption to accommodate uneven magnitudes of selective pressures ($|s_{1} | \ne$ $|s_{2} |$) and random perturbations of the selection coefficient by replacing the *s*_*i*_ above with $\left( {1 + \varepsilon_{t} } \right)s_{i}$, where $\varepsilon_{t}$ is drawn independently in each generation from *N*(0, $\sigma$).

A plasticity effect, *p*_*hi*_, represents the net effects of benefits and costs of the modifier allele for a specific target allele *h* in a specific deme *i*. We assume that the benefit of plasticity exceeds the cost and increases the fitness of its carriers when the *M* allele is paired with a maladapted target allele (*d* in deme 1 and *a* in deme 2). When the *M* allele is paired with the adapted target allele that is already near the fitness optimum, the cost of the plasticity exceeds the benefit resulting in a relative decrease in the fitness of its carriers. These fitness values results in the plasticity modifier allele being disadvantageous (maladaptive) in the ancestral deme when cost is present (cost > benefit; $\frac{{w_{aM1} }}{{w_{am1} }} < 1$), but advantageous in the derived deme (benefit > cost; $\frac{{w_{aM2} }}{{w_{am2} }} > 1$) if the population is monomorphic for the ancestral allele. This situation occurs if costs, such as might arise due to maintenance of plasticity structures, are independent of the environment and therefore constant across environments whereas the benefit only occurs in adverse environments. First, we model a symmetric case in which *s*_*i*_ =  ± *s* and *p*_*a1*_ = *p*_*d1*_ = *p*_*a2*_ = *p*_*d2*_ = *p*. This case is equivalent to assuming that an environment-specific benefit to maladapted target alleles *b* is two times the environment-independent cost *c*, i.e. *b* = 2*c*. In this scheme, plasticity moves phenotypes towards the middle of the range of possible fitnesses across demes. In this symmetric situation, neither the net cost nor benefit to plasticity are overwhelming, which might otherwise drive the plasticity modifier to either fixation or loss. We later relax this assumption to accommodate a wider range of implied benefit and cost relationships (Supplementary Information 2 Fig. S1 online).

During periods when a deme is polymorphic at both loci, the expected haplotype frequencies, $x_{{{\text{ami,}}t}}^{\prime } , x_{{{\text{aMi,}}t}}^{\prime } , x_{{{\text{dmi,}}t}}^{\prime } ,\;{\text{and}}\;x_{{{\text{dMi,}}t}}^{\prime }$ at time *t* are also altered by recombination. With the coefficient of linkage disequilibrium, $D_{i,t} = x_{{{\text{ami,}}t}}^{\prime } x_{{{\text{dMi,}}t}}^{\prime } - x_{{{\text{aMi,}}t}}^{\prime } x_{{{\text{dmi,}}t}}^{\prime }$, the expected frequencies of four haplotypes following recombination are given by

6 $$x_{{{\text{ami,}}t}}^{\prime \prime } = x_{{{\text{ami,}}t}}^{\prime } - D_{i,t} r,$$

7 $$x_{{{\text{aMi,}}t}}^{\prime \prime } = x_{{{\text{aMi,}}t}}^{\prime } + D_{i,t} r$$

8 $$x_{{{\text{dmi,}}t}}^{\prime \prime } = x_{{{\text{dmi,}}t}}^{\prime } + D_{i,t} r$$

and

9 $$x_{{{\text{dMi,}}t}}^{\prime \prime } = x_{{{\text{dMi,}}t}}^{\prime } - D_{i,t} r$$

where *r* is the recombination rate between the two loci. When a meta-population is monomorphic at the target locus, recombination is expected to have no effect on evolution at the plasticity locus.

To account for genetic drift in finite populations, we sample *N*_*i*_ individuals for the next generation in each deme *i* from a multinomial distribution using the expected haplotype frequencies $x_{{{\text{ami,}}t}}^{\prime \prime } , x_{{{\text{aMi,}}t}}^{\prime \prime } , x_{{{\text{dmi,}}t}}^{\prime \prime } ,\;{\text{and}}\;x_{{{\text{dMi,}}t}}^{\prime \prime }$ (Eqs. 6–9), resulting in sampled frequencies in the subsequent generation, namely $x_{{\text{ami,t + 1}}} ,\; x_{{\text{aMi,t + 1}}} ,\;x_{{\text{dmi,t + 1}}} ,\;{\text{and}}\;x_{{\text{dMi,t + 1}}}$.

### Inferring long-lived balanced polymorphism

Diversity is a proxy for the potential for the rapid molecular evolution of phenotypic plasticity. We, therefore, focus on the presence and stability of balanced polymorphism. We measure diversity levels as the expected cumulative heterozygosity over time^53^ at the plasticity modifier locus. Since population structure can exaggerate the level of diversity, we measure the expected cumulative heterozygosity within a deme, averaged over the meta-population^27^,

$${\rm H}_{{\text{L}}} = 2\left[ {\mathop \sum \limits_{t} \mathop \sum \limits_{i} \frac{{x_{Mi,t} \left( {1 - x_{Mi,t} } \right)N_{i} }}{N}} \right].$$

In this formula, $x_{Mi,t}$ is the plasticity modifier allele frequency after migration, selection, and reproduction in deme *i* at time *t*, $N_{i}$ is the size of deme *i*, and *N* = *N*_1_ + *N*_2_ is the size of (meta-) population. The cumulative heterozygosity *H*_L_ measures diversity over time. Under drift or directional selection, alleles are fixed or lost over time and *H*_L_ approaches a finite expectation with increasing simulation time. Under drift, this expectation is 2 irrespective of population size^53^ and population structure^27^. In cases of balanced polymorphism, however, heterozygosity persists for long times, making *H*_L_ roughly proportional to time even for long simulations, up until the point that alleles go extinct due to long, improbable excursions from equilibrium. This cumulative heterozygosity thus reflects not only the plasticity modifier allele frequencies over time but also the strength of stability of the equilibrium against stochastic fluctuations. Observing *H*_L_ >  > 2 over a long simulation time, or observing *H*_L_ continuing to increase over long times therefore proves the presence of long-lived stable polymorphic equilibria. We therefore use *H*_L_ to confirm the existence of intermediate-frequency stable equilibrium predicted by our stability analysis (Supplement 1). Note that *H*_L_ conflates the equilibrium allele frequency, its stability over time, and the probability for newly introduced mutations to reach equilibrium before going extinct due to random drift. *H*_L_ >  > 2 nonetheless indicates conditions supporting long-term persistent diversity and therefore the potential for rapid evolution.

### Simulations

We simulate evolution in populations starting from an initial mutation until either the metapopulation reaches monomorphy through fixation or loss of the mutant alleles or else polymorphism persists for 100*N* generations, where *N* is a metapopulation size. Depending on population sizes (see below), therefore, simulations were run for between 40,000 and 2 million generations, way beyond the expected lifetime of a neutral polymorphism, to assure the detection of long-lived stable polymorphism. We conduct many replicates because most single mutant introductions in finite populations are lost to stochastic extinction^54^. We therefore conduct 1000*N* replicates for each combination of local directional selection effects (*s* = 0.0, 0.005, 0.01, 0.015, 0.02, 0.025, 0.03), migration rates (*e*_1_ = 1, 5, 10, 25, 50, 75, 100, 250, 500, 750, 1000, 1250, 1500, 1750, 2000, 2250, 2500, 2750, 3000, 3250, 3500, 3750, 4000, 4250, 4500, 4750 5000, *e*_2_ = *e*_1_ or 0), the period between migration events (*I* = 1, 10, 25, 50, 100, 200, 400, 500, 600, 800, 1000), plasticity effects (*p* = 0.25, 0.5, 0.75, and 1), and recombination rates (*r* = 0.0001, 0.01, 0.1, 0.25, 0.5), with or without environmental perturbations ($\sigma$ = 0, 0.1, 0.3. and 0.5). We test all parameter combinations in the large equal-sized populations *(N*_1_ = *N*_2_ = 10,000, 1000, or 200), and a subset of combinations in unequal populations where the ancestral deme is much larger (*N*_1_ = 10,000 or 1000) than the derived deme (*N*_2_ = 2000 or 200), with the mutation occurring only once per simulation run at each of the loci with probability = 0.001, 0.1, or 1.0 in each generation.

## Results

**Figure 1:**
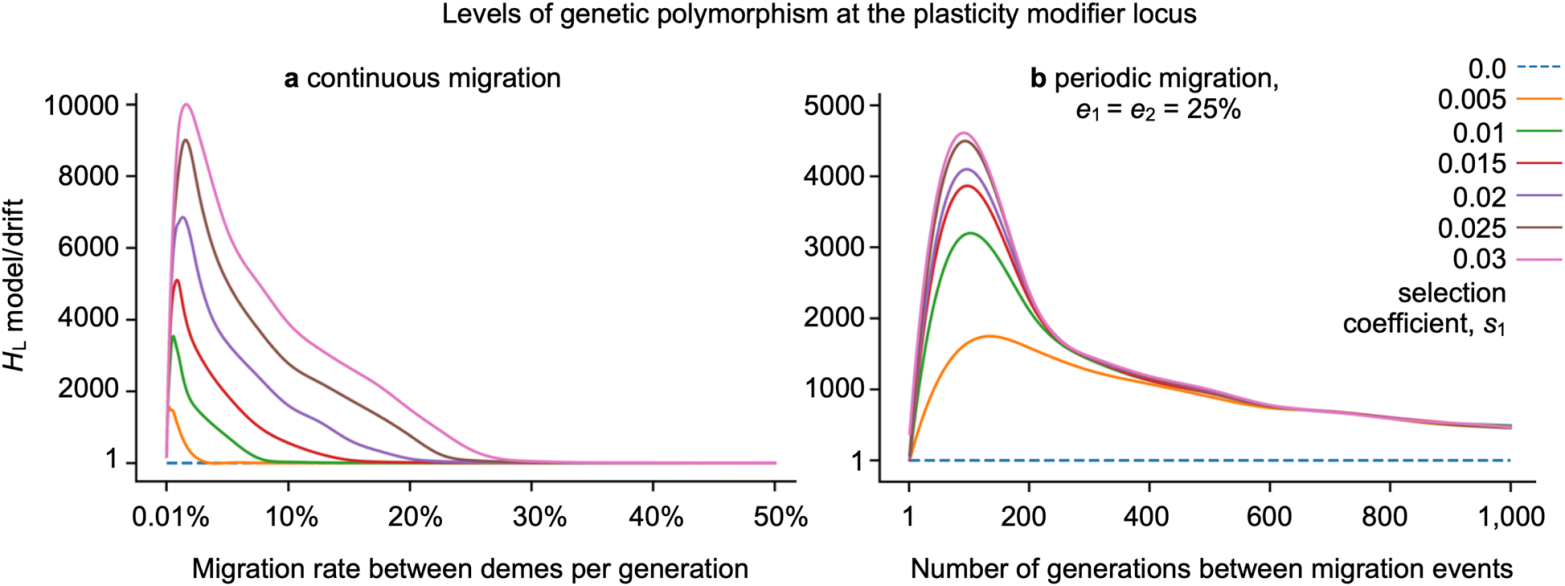
Balanced polymorphism at the plasticity modifier locus is driven by population structure in patchy habitats. The levels of cumulative heterozygosity at the plasticity modifier locus (> 1 exceeds neutrality, broken line) in structured populations with (**a**) migration between habitat patches each generation, i.e. continuous migration, *I* = 1, or (**b**) migration occurring periodically, each *I* generations, here with one-quarter of the population exchanging residence, across a range of symmetrically opposing selective pressures (*s* = *s*_1_ =  − *s*_2_ = 0.0, 0.005, 0.01, 0.015, 0.02, 0.025, 0.03). Forward-in-time computer simulation shown here assumed a metapopulation with two equal-sized populations (demes) of *N*_i_ = 10,000 individuals and were conducted over 2 million generations and 20 million replicate runs per parameter combination where the symmetric plasticity effect, *p* = 1.0 and recombination rate *r* = 0.5. The curves were smoothed using a spline function and perfectly align with the data points.

**Figure 2:**
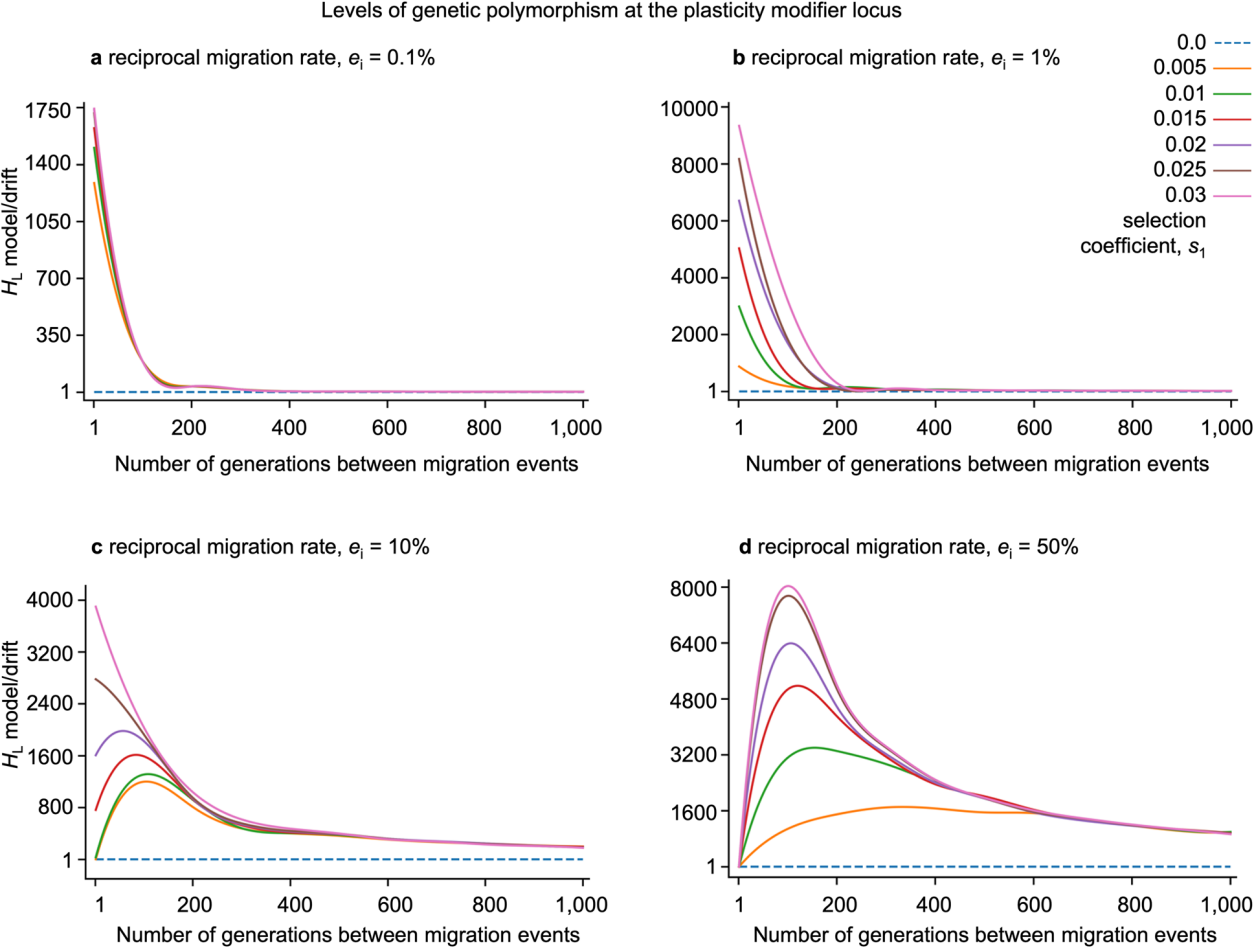
Balanced Polymorphism at the Plasticity Modifier Locus Arises Under Periodic Migration. The levels of cumulative heterozygosity at the plasticity modifier locus (> 1 exceeds neutrality, broken line) in structured populations with reciprocal migration with (**a**) *e*_*i*_ = 10 (0.1% of a deme) (**b**) *e*_*i*_ = 100 (1% of a deme) (**c**) *e*_*i*_ = 1000 (10% of a deme) and (**d**) *e*_*i*_ = 5000 individuals (50% of a deme) migrating between habitat patches once each *I* generations. 5000 individuals (**d**) represents free migration known as “panmixia”. Simulations were conducted across a range of symmetric opposing selective pressures (*s* = *s*_1_ =  − *s*_2_ = 0.0, 0.005, 0.01, 0.015, 0.02, 0.025, 0.03). Forward-in-time computer simulation shown here assumed a metapopulation with two equal-sized populations (demes) of *N*_i_ = 10,000 individuals and were conducted over 2 million generations and 20 million replicate runs per parameter combination where the symmetric plasticity effect, *p* = 1.0 and recombination rate *r* = 0.5. The curves were smoothed using a spline function and perfectly align with the data points. Note different scales of y-axes.

**Figure 3:**
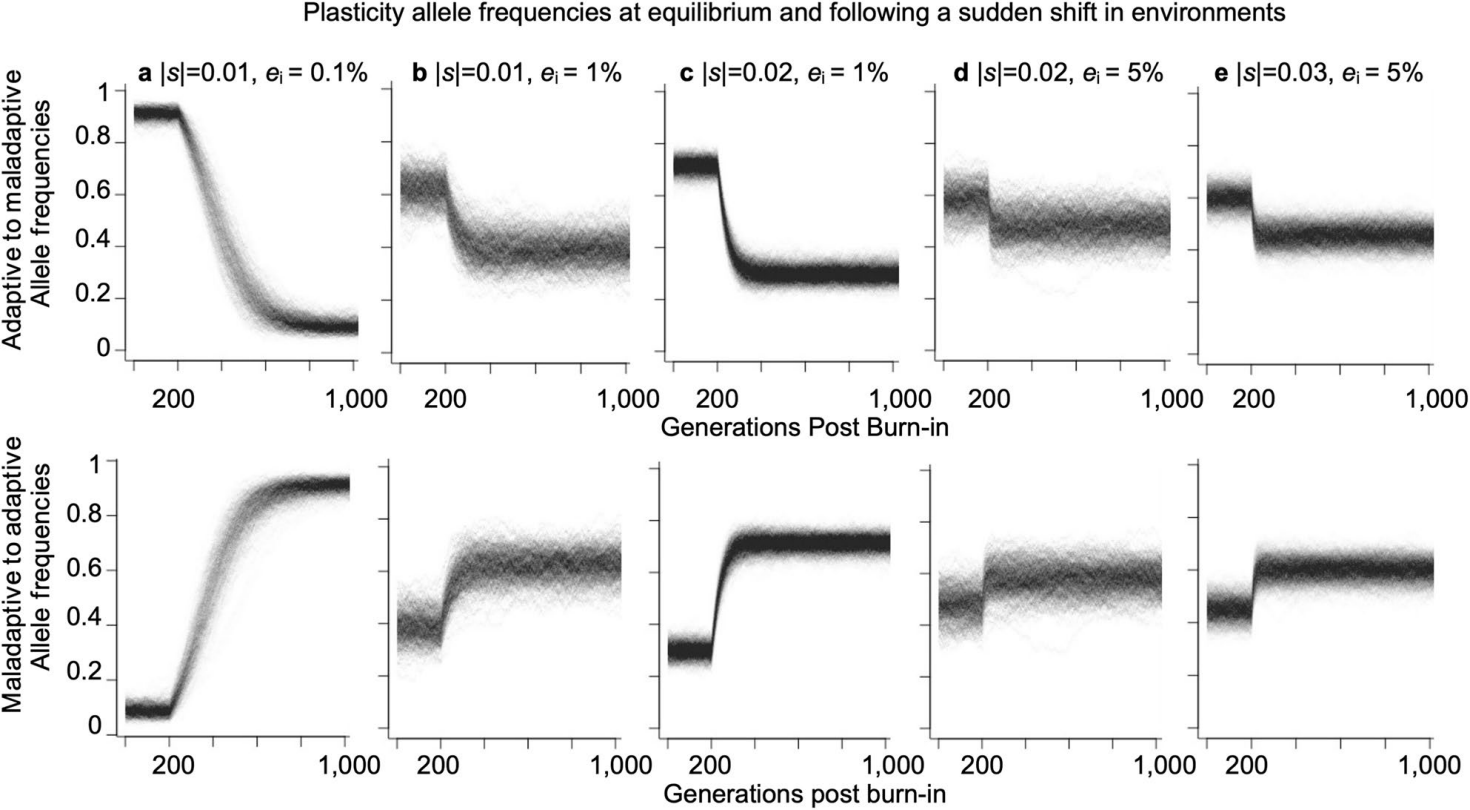
Balanced polymorphism promotes rapid evolution of phenotypic plasticity under environmental change. Plasticity modifier allele frequencies over 200 generations starting at quasi-equilibrium (following 800 thousand, 40*N*, generations of burn-in) in the derived deme (top row) and ancestral deme (bottom row), followed by 800 generations after the sudden switch in environments. (**a**) 485 runs resulting in the polymorphic plastic locus observed with |*s*|= 0.01 and 10 migrants (0.1% of a deme) per generation, (**b**) 338 runs resulting in the polymorphic plastic locus observed with |*s*|= 0.01 and 100 migrants (1% of a deme) per generation, (**c**) 809 runs resulting in the polymorphic plastic locus observed with |*s*|= 0.02 and 100 migrants (1% of a deme) per generation, (**d**) 367 runs resulting in the polymorphic plastic locus observed with |*s*|= 0.02 and 500 migrants (5% of a deme) per generation, and (**e**) 767 runs resulting in the polymorphic plastic locus observed with |*s*|= 0.03 and 500 migrants (5% of a deme) per generation. Simulations assumed a large population of *N*_*i*_ = 10,000 individuals and were conducted over 20 million replicate runs with per parameter combination where the symmetric plasticity effect, *p* = 1.0, and recombination rate *r* = 0.5.

**Figure 4:**
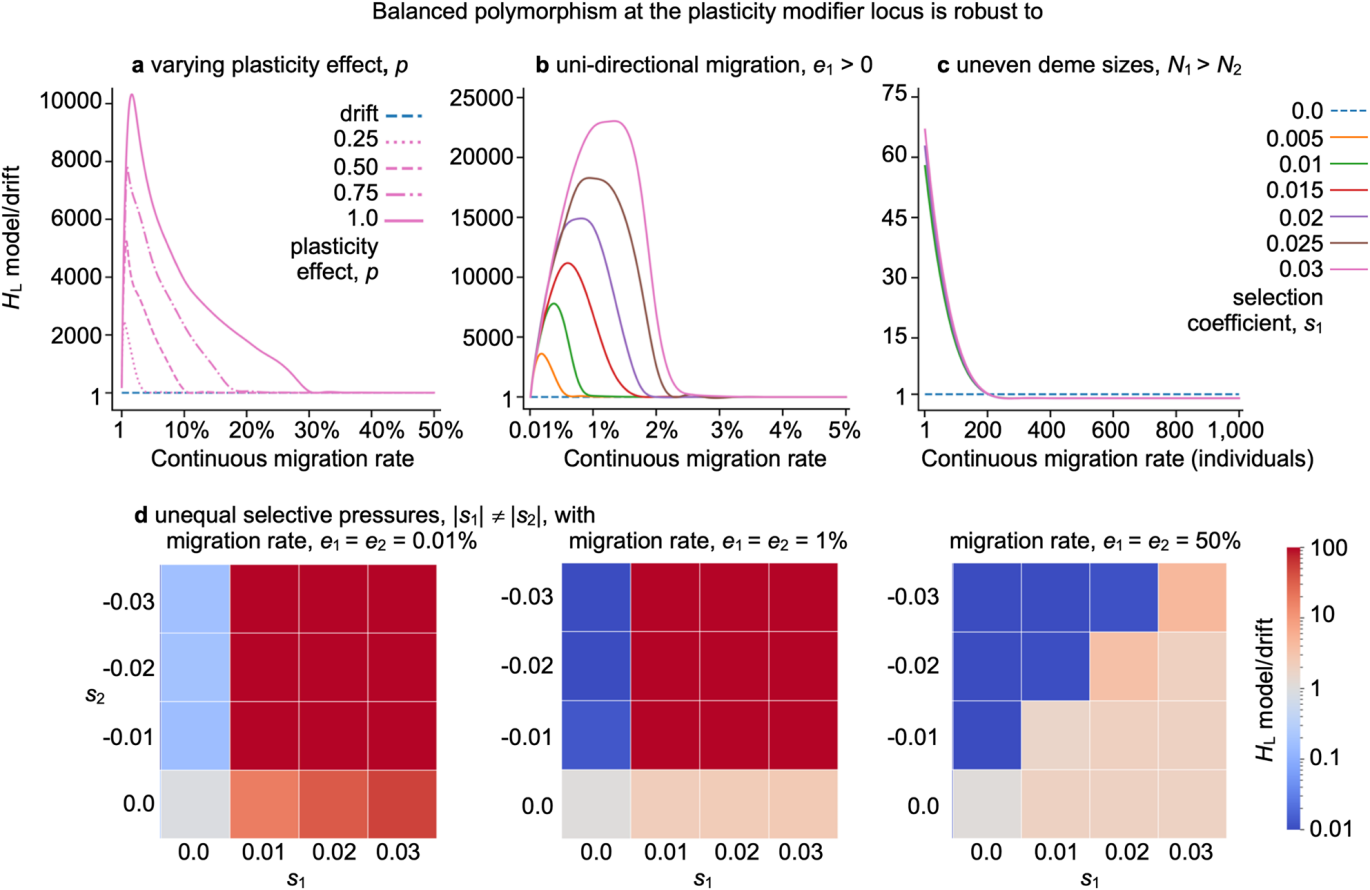
The plasticity-balancing effect of population structure in patchy habitats is robust to relaxing simplifying assumptions. The levels of cumulative heterozygosity at the plasticity modifier locus (> 1 exceeds neutrality, broken line) in structured populations when (**a**) symmetric plasticity effect, *p* = 0.25, 0.5, 0.75, or 1.0, shown with opposing selective pressures $s = s_{1} = - s_{2} =$ 0.03, (**b**) only the derived deme receives a single plastic mutant and immigrants (polymorphism only measured in derived deme) with varying symmetric opposing selective pressures ($s = s_{1} = - s_{2} =$ 0.0, 0.005, 0.01, 0.015, 0.02, 0.025, 0.03), with both *N*_1_ = *N*_2_ = 10,000, and (**c**) when the derived deme is much smaller in size than the ancestral deme with *N*_1_ = 10,000 and *N*_2_ = 2000 individuals, with various opposing selective pressures $s = s_{1} = - s_{2}$ or (**d**) when there are different magnitudes of opposing selective pressure acting in each deme, with 1 (0.01% of a deme), 100 (1% of a deme), or 5000 (50% of a deme) reciprocal migrants changing residence each generation. Forward-in-time computer simulations were conducted over 2 million generations and 20 million replicate runs per parameter combination where recombination rate *r* = 0.5. The curves were smoothed using a spline function and perfectly align with the data points.

In silico, we observe a wide range of naturally plausible scenarios where balanced polymorphism at the plasticity modifier locus arises in both the ancestral and derived deme, as long as there is population structure due to limited migration (gene flow) between the demes (Figs. 1, 2, 3, 4). We recover balanced polymorphism in plasticity with periodic and continuous migration events between habitat patches (Figs. 1b, 2), bidirectional and unidirectional migration between demes (Fig. 4), symmetric and uneven magnitude of selective pressures in the two demes (Fig. 4), and with symmetric and asymmetric or small deme sizes (Fig. 4, Supplemental Fig. S2 online), and across varying degrees or asymmetry of relative fitness effects of plasticity (Fig. 4, Supplemental Fig. S3 online). Since balanced polymorphism promotes the potential for a rapid plastic response, these results suggest that phenotypic plasticity can quickly evolve across a wide spectrum of naturally realistic scenarios in response to sudden environmental change, provided that there is population structure and heterogenous selection across populations, such as across a patchy habitat. For example, following a sudden switch in a deme’s adaptedness, such as might arise from changing climate, range shift of predators or competitors, local introduction of invasive species, or local adaptation in derived deme, we show that the equilibrial frequency of plastic phenotypes readily and rapidly changes and stabilizes at a new level (Fig. 3).

Mathematical analysis described in Supplementary Information 1 confirms that the persistent polymorphisms observed in stochastic simulations correspond to stable equilibria in infinite populations with deterministic dynamics, indicating balanced polymorphisms maintained by selection, plasticity, and population structure. We analyze the symmetric case in which $e = e_{1} = e_{2}$, $s = s_{1} = - s_{2}$, and *p* = *p*_*a*1_ = *p*_*d*1_ = *p*_*a*2_ = *p*_*d*2_. The result is that intermediate equilibria representing balanced polymorphism exist in both ancestral and derived demes and are stable whenever *sp* and *e*/*IN* are strictly greater than zero. That is, the deterministic condition for stable polymorphism requires migration, selection, and some net effect of cost and benefit to plasticity and is fulfilled whenever migration between demes (*e*/*I*), selection (*s*), and the effect of plasticity (*p*) are all finite and greater than zero. For high migration rates, however, the equilibria collapse onto a line representing homogenization and loss of structure between the two demes. Then, the plasticity modifier either fixes or goes extinct in the limit of very large migration rates depending on its adaptive status. This effect highlights that limited migration is required for the protection of balanced polymorphism. The equilibrium frequencies of the *M* allele in deme *i*, $x_{i}^{*}$, depend only on the relative strength of selection (*sp*) and migration (*e*/*IN*) in the deterministic model:

$$x_{1}^{*} = \frac{1}{2}\left( {1 + \frac{\cos \left( \theta \right) - 1}{{\sin \left( \theta \right)}}} \right), x_{2}^{*} = x_{1}^{*} \frac{\sin \left( \theta \right) + 1}{{{\text{cos}}\left( \theta \right)}}, \theta = {\text{tan}}^{ - 1} \left( {\frac{sp}{{e/IN}}} \right).$$

We can represent the relative strength of selection and migration with the parameter $\theta$ between 0 (no selection) and $\pi$/2 (no migration). This parameterization makes it possible to discern from the formulas that $x_{1}^{*}$ is bounded between 0 and ½, whereas $x_{2}^{*}$ is bounded between ½ and 1 (cf. Supplementary Information 1), and so the equilibria exist in the interior of the frequency range for typical parameter values that involve some amount of migration and selection.

### Balanced polymorphism at the plasticity modifier locus is driven by population structure in patchy habitats

Here we describe in more detail how population structure drives balanced polymorphisms in plasticity and how robust the described effects are to changes in parameters and perturbations in the model settings. In particular, we observe balanced polymorphism at the plasticity modifier locus as evidenced by levels of cumulative heterozygosity (*H*_L_) exceeding that expected under neutrality (and directional selection), across a range of rates and types of migration. Balanced polymorphism exists for limited continuous reciprocal rates and periodic reciprocal migration, provided there is some degree of population structure (Figs. 1, 2). If the migration is continuous, we observe slightly elevated polymorphism with low migration rates (e.g. *e*_1_ = *e*_2_ = 1 migrant per generation) with higher levels of balanced polymorphism across moderate migration rates, with some increase in diversity for migration rates up to 20% of a deme’s residents per generation (Fig. 1a). The polymorphism-promoting effect of population structure is lost when reciprocal migration rates exceed 20% of a deme (Fig. 1b), unless migration is limited to periodic occurrences where balanced polymorphism is observed even under occasional panmixia (Fig. 2). Therefore, population structure, due to limited rates or limited periods of migration, is necessary to protect the locally adaptive alleles at the plasticity modifier locus in finite populations. Because *H*_L_ combines the equilibrium frequency, long-term persistence of alleles, and probability for a single mutant to reach equilibrium before stochastic extinction, we also disentangle these effects by examining how the frequency of the stable equilibrium and the probability of avoiding stochastic extinction depend on migration (Supplemental Fig. S4 online). As predicted in the deterministic analysis, equilibrium frequencies interpolate between the boundaries and ½ depending on the migration rate. In any finite population, the vast majority of new mutants go extinct quickly and do not persist, even when a mutant is positively selected^54–56^. We observe rates of persistence of the same order as positive selection and orders of magnitude higher than under drift.

Sufficiently high migration rates erase population structure in the symmetric, deterministic analysis as well (Supplemental Information 1). As migration rates increase, the interior equilibria in each deme approach one another and stability weakens, as we also observe in simulation (Fig. S4). When *e*/*IN* becomes infinite, there is a line of neutrally stable equilibria where genotype frequencies are identical in both populations. At this point, because the selection is symmetric and essentially random, the metapopulation evolves as a single neutral population and alleles are fixed or lost by drift. Evolution in the limit of large migration rates (homogenized population) becomes non-neutral if there is a net adaptive or maladaptive effect to plasticity over both environments, in which case the balance is broken and plasticity allele is selected to either fixation or extinction. This highlights, again, that population structure is needed for the protection of polymorphism across patchy habitat.

Diversity levels increase with selection pressure on the target locus, such that under weaker selection regimes, balanced polymorphism depends on the more rigid population structure that occurs with lower migration rates in comparison to when selection pressure is stronger (Fig. 1). It is worth noting that selective pressure on the target locus under our model is proportional to the benefit of plasticity in the harmful habitat patch and to the cost of plasticity in the beneficial habitat patch. Hence, stronger selection assures protection of the locally adapted plasticity modifier allele even when high migration rates allow strong perturbations to its frequency. This result is consistent with the deterministic analysis in which the equilibrium genotype frequencies depend only on the ratio of selection to migration, *spIN*/*e*. Thus, proportionately stronger selection can buffer against genetic swamping due to higher migration.

In the case of periodic migration, diversity levels strictly increase with rates of migration provided migration is sufficiently rare and decrease monotonically with the number of generations between migration events when the migration rate is low (Figs. 1b, 2). For sufficiently rare migration events, the level of *H*_L_ becomes independent of the strength of selection. Remarkably, with high migration rates per event even including panmixia, (Figs. 1b, 2c,d) we recover balanced polymorphism at the plasticity locus even when migration occurs very rarely, such as only once every 1000 generations. These results imply that isolated populations can maintain polymorphism in plasticity for a long time and retain the ability to rapidly acclimate to new conditions if they have experienced substantial gene exchange with another deme under a different selection regime in the past.

### Balanced polymorphism promotes rapid evolution of phenotypic plasticity under environmental change

To confirm that rapid adaptation at the plasticity locus can arise from balanced polymorphism, we induced a sudden environmental change in the simulation by swapping the selective environment across the two demes. We observed the evolutionary response to this sudden shift by measuring the plasticity modifier allele frequencies for 200 generations at quasi-equilibrium. That is, following 800 thousand (40*N*) generations of burn-in simulation, which exceeds 10 times neutral expectation in fixation times and therefore demonstrates a long-lived stable polymorphism, we induced an environmental switch after 200 generations by changing the sign of the selective coefficients at 40*N* + 200 generations, and then observed the simulation for the next 800 generations. Following the switch in demes’ selective environments, plasticity modifier allele frequencies rapidly decrease in the derived deme when it becomes adapted and show the mirror opposite pattern in the ancestral deme when it becomes maladapted (Fig. 3).

In particular, when the migration rate is low (Fig. 3a), the plasticity modifier allele stabilizes at a high frequency in the maladapted derived deme and at a low frequency in the adapted ancestral deme, in accordance with plasticity being adaptive in one and maladaptive in the other. As conditions switch resulting in the opposite directional selection, both demes rapidly evolve to the previous equilibrial frequency of the opposite deme. The rate of rapid evolution increases with heterozygosity (Fig. 3b vs. a): adaptation on plasticity to the new equilibrial frequency occurs more quickly when the equilibrium frequency balanced polymorphism is closer to 1/2. At such higher levels of balanced polymorphism, modifier allele frequencies are maintained at more intermediate values, in contrast to cases of lower balanced polymorphism where the allele frequencies are maintained closer to the frequency boundaries 0 or 1. Stronger selection pressure (Fig. 3c vs. b and e vs. d) pushes equilibrium frequencies marginally closer to the boundaries, whereas higher migration rates quickly bring the equilibria closer together, as expected from the deterministic analysis. Closer equilibria correspond to faster evolution at the plasticity locus following an environmental switch, indicating that migration rates are the major determinant of both the levels of balanced polymorphism and consequently of rates of rapid adaptation at the plasticity locus, across our parameter range.

Overall, these results show that plasticity polymorphism results in both demes rapidly evolving to new equilibrium frequencies following an environmental shock, across the range of equilibrial allele frequencies. Therefore, rapid adaptation is expected to occur if plastic alleles are present in the population, indicated across a broad range of parameters where elevated *H*_L_ is observed. Moreover, both demes retain balanced polymorphism for plasticity as long as there is migration between selectively distinct habitats. These results, therefore, offer a plausible natural scenario for the rapid evolution of plasticity in populations in patchy habitats, where plasticity aids in population persistence and affords the capacity for rapid evolution in response to changing environmental conditions.

### The plasticity-balancing effect of population structure in patchy habitats is robust to changes in model assumptions

Next, we tested how perturbations in the simplifying assumptions, such as symmetric deme sizes, symmetric magnitudes of selection pressure, symmetric migration, the degree and symmetry of plasticity effect, and large census meta-population size affect the maintenance of balanced polymorphism in structured populations. We find that the qualitative effect still occurs under some range of parameters (Fig. 4, Supplementary Figs. S2 and S3 online). While the levels of balanced polymorphism tend to decrease with the effect of plasticity, as expected, balanced polymorphism is still observed even with *p* = 0.25 but with lower migration rates (Fig. 4a), consistent with the deterministic finding that equilibria depend on the ratio of *sp* and *e*/*IN*. Again, when the plasticity effect is weaker (ameliorates the effect of selection to a small degree), its polymorphism requires protection by limited migration and limited opportunities for one population to genetically swamp the other.

We postulate that the balanced polymorphism is maintained in each deme by the balance between limited relative immigration (proportion of post-deme membership originating from another deme) and selection on the plasticity locus, and does not depend on reciprocal emigration rate. For example, while selection prefers the plasticity modifier allele in the derived deme, limited immigration from the ancestral deme prevents its fixation without swamping the derived deme even for unidirectional migration. Conversely, the opposite occurs for the ancestral deme if migration occurs in the opposite direction. Therefore, as long as there is limited relative immigration to a deme, even by unidirectional migration, we expect polymorphism in the recipient deme (Fig. 4b). Indeed, balanced polymorphism in the derived deme persists provided immigration rates are limited, as migration serves only to reintroduce non-plastic allele copies. These results indicate that the balancing effect of population structure and patchy habitats on the plasticity locus applies to the source–sink and pseudo-source–sink dynamics^50–52^ as well.

An implication of the dynamics above is that, in the case of unidirectional migration, the cost of plasticity is inconsequential if plasticity is adaptive in adverse environments. That is, genetic polymorphism in the adverse environment depends on the balance between positive selection on adaptive plasticity and immigration of non-plastic phenotypes from the favorable environment, irrespective of whether the ancestral deme is non-plastic due to plasticity being maladaptive (costly) or neutral (not costly).

Next, we examined a scenario where the derived deme is much smaller than the ancestral deme. Here, we find that migrants from the ancestral deme quickly genetically swamp the smaller deme if migration rates are high and that this obliterates the polymorphism at the plasticity locus (Fig. 3c). Yet, lower migration rates (up to 20% of *N*_2_) still permit high levels of balanced polymorphism. In other words, plasticity is maladaptive for the majority of individuals in the metapopulation who live in the ancestral deme, and so in the absence of structural protection in the smaller deme, the polymorphism at the plasticity locus is removed quickly to levels below that under drift (Fig. 4c). However, the overall balancing effect of population structure is present even when the non-adapted population is small, provided limited migration.

While above we examine the evolution of phenotypic plasticity in a large meta-population, as might arise due to split in large natural populations or due to large invasive demes created by global shipping transport, we confirm the balancing effect in small demes (Supplementary Fig. S2 online). The qualitative patterns of balanced polymorphism described in large populations are observed in small populations, more so with stronger selection, but under smaller migration rates. With equal deme sizes *N*_1_ = *N*_2_ = 1000 the effect holds with up to 25% deme changing residence as in a large population (Supplementary Fig. S2a online), but once deme sizes are reduced to *N*_1_ = *N*_2_ = 200 the weaker effect is observed with up to 10% residents changing demes, less so with weaker selection and higher migration rates (Supplementary Fig. S2b online). When the ancestral deme is larger than the derived deme with *N*_1_ = 1000 and *N*_2_ = 200, we observe similar patterns of balanced polymorphism as in the larger unequal demes, but again over a narrower range of migration rates up to 5% of *N*_2_ (Supplementary Fig. S2c online). With stronger genetic drift, the balancing effect in patchy habitats requires a stronger population structure.

The balancing effect of population structure on phenotypic plasticity is robust to asymmetry in the magnitude of selective pressures in the two demes as well. Figure 4d indicates that high levels of diversity due to balanced polymorphism occur with asymmetric selection as long as selection is of opposite direction in the two demes and migration is limited. The effect is lost with a high migration rate, as observed in the symmetric case (Fig. 1), due to the disappearance of population structure. With a panmictic population, if the magnitude of selection is larger in the derived than ancestral deme, phenotypic plasticity carries more benefit to the derived deme than its cost to the adapted deme resulting in it being overall beneficial with a slightly elevated cumulative heterozygosity, *H*_L_ < 4, due to a higher chance of fixation of the plasticity allele, as observed in other studies^13,27^ (Fig. 4d right panel). This, however, does not signify balanced polymorphism as selection becomes directional and acts only to fix the plasticity modifier allele, not to protect against fixation or loss for both alleles.

Notably, allowing random perturbations to the selection coefficients did not qualitatively affect the levels of balanced polymorphism under population structure and patchy habitats, producing plots that are not visually distinguishable from those in Fig. 1a.

For the results presented so far, we have assumed that the rates of recurrent mutation are low, while we still account for the possibility of interaction between the target and plasticity loci by simulating random single-mutant introduction at each locus. Indeed, in cases where constitutive adaptation occurred in the derived deme, plasticity was then selected out. These events were rare, however, and are accounted for in the reported levels of polymorphism. Consequently, the interaction between adaptive dynamics at the two loci has a small effect on *H*_L_. By disallowing adaptation at the target locus (example in Supplementary Fig. S5 online) we observe that the effect of interference is merely a few percent reduction in diversity, which we confirm across the range of migration rates resulting *H*_L_ >  > 2. Therefore, when recurrent mutation is rare, the results approximate cases where constitutive adaptation is not possible, effectively producing single-locus dynamics for the plasticity modifier allele.

If we allow recurrent mutation at the plasticity locus (Supplemental Fig. S5 online bottom row), each mutant introduction has a chance to reach the stable intermediate frequency. Under recurrent mutation, new mutants enter the population at a rate of $N\mu$ per generation. Therefore, the rate of at least one mutant reaching equilibrium increases over time roughly in portion to $N\mu$ (Supplemental Fig. S5c online bottom panel). Recurrent mutation thus also further elevates cumulative heterozygosity relative to drift (Supplemental Fig. S5a online bottom panel). Recurrent mutation also allows more chances for adaptation at a target locus, however, and therefore more interference between the two loci arises. In this case, the discrepancy between the single- and two-locus dynamics increases over time (Supplemental Fig. S5 online bottom row).

Since simultaneous polymorphism at the two loci was not likely, recombination did not have an observable effect on evolution at the plasticity locus. Therefore, the results above generalize across recombination rates from cases with no recombination, which fall under the environmentally sensitive loci model of phenotypic plasticity, to cases of epistatic plasticity modifier loci that are linked or unlinked to the target (structural) locus, making the above results applicable regardless of assumptions about the genetic architecture and specific mechanisms of phenotypic plasticity.

In the cases where adaptation at the target locus occurred in the adverse habitat patch, the plasticity modifier allele was selected out if the meta-population evolved towards balanced polymorphism at the target locus, driven by adaptation in each deme and migration between demes. In the absence of recurrent mutation, we do not observe any notable effect of the rate of introducing a new mutant to either locus, on the patterns of molecular evolution at either locus.

We also initially assumed symmetric fitness effects of plasticity in order to produce intermediate fitnesses where the adaptive value of plasticity in adverse habitats would not greatly exceed the maladaptive effects in favorable habitats or vice versa. Once the adaptive effects of plasticity and maladaptive effect become asymmetric balance in each deme is perturbed and allele frequencies may approach the boundaries 0 and 1. In finite populations this proximity to the boundaries makes fixation likely. Indeed, in the range of asymmetric fitness effects involving a small cost, including no cost, the balancing effect is limited to stronger population structure. That is, we observe elevated diversity in the limit of lower migration rates (Supplementary Figs. S1 and S3 online). When costs are small, the balancing effect is overwhelmed by adaptive fixation of the plasticity modifier allele since the benefit far exceeds the cost across a range of sufficiently high migration rates, including panmixia (Supplementary Fig. S3). Therefore, plasticity is expected to rapidly evolve due to balanced polymorphism across the range of plasticity effects if there is adaptive plasticity in a derived deme and some cost in the ancestral deme. The effect is strongest with the symmetric fitness effects of plasticity across patchy habitats and depends on stronger structure when plasticity effects are asymmetric and costs of plasticity are small or negligible (Supplementary Fig. S3 online).

Another consequence of asymmetry is that diversity levels may differ between populations (Fig. S3 online bottom row). Under symmetric plasticity and selection effects, the observed metapopulation cumulative heterozygosity *H*_L_ and those in each deme are nearly equal, and so can be interpreted interchangeably. In asymmetric cases, however, we maintain that metapopulation *H*_*L*_ still indicates the possibility for rapid adaption since evolution occurs readily across the range of starting allele frequencies provided that adaptive alleles are present in a population at frequencies away from the boundaries 0 and 1 (Fig. 3). Even if balanced polymorphism for plasticity is maintained in only one deme, migration is far more likely to introduce the modifier allele to the other deme following an environmental shift in comparison to adaptation that relies on de novo beneficial mutation. Metapopulation diversity necessarily summarizes information and maintains relevance for rapid adaptation.

## Discussion

This study demonstrates a novel mechanism for the rapid evolution of phenotypic plasticity: genetic variation in phenotypic plasticity maintained by balancing selection in patchy habitats. We propose that populations that have previously experienced limited immigration from habitat patches where plasticity had an opposing selective effect may harbor balanced polymorphism for plasticity. These populations are then potentiated to rapidly evolve plasticity when the environment changes suddenly, for example with climate change, range shift of symbionts, sudden environmental shifts in patches within a habitat matrix, or invasion of a subpopulation into a new habitat. This is because once alleles are present at a stable intermediate frequency, allele frequencies quickly approach new equilibria in response to changes in selection or migration between demes. Not only can the proposed mechanism explain the rapid evolution of plastic response in invaders from favorable habitats, but it also suggests that populations in adverse habitats will maintain a sizable proportion of non-plasticity alleles and be able to rapidly evolve non-plastic phenotypes if constitutive adaptation occurs, known as assimilation^57,58^.

We report a mechanism by which plasticity modifier alleles can achieve long-term balanced polymorphism at rates comparable to directionally-selected alleles following single-mutant introduction. The actual prevalence of loci under balanced polymorphism in wild populations due to this mechanism in turn depends on rates of introduction of plasticity modifier alleles, migration rates, and the history of selective conditions across populations. This helps explain how, given the right conditions, genetic diversity that supports rapid evolution of plasticity can accumulate and be maintained in populations over time.

The observed effect depends on population structure due to either limited continuous rates of migration or limited frequency of migration events, and on spatially heterogenous indirect selection on the plasticity locus. The effect still occurs in cases of reducing the plasticity effect, introducing uneven selective pressures, uneven or small population sizes, perturbations to the selection coefficient or asymmetry in plasticity effects, and across a wide range of migration rates and periods of complete isolation between demes. Moreover, the results are robust to the recombination rates between the plasticity modifier and the target gene, making them broadly applicable across models of the genetic basis of phenotypic plasticity. That is, the effect appears equally in environmentally sensitive loci and epistatic modifier loci, since the difference between these bases of phenotypic plasticity is in whether there is recombination between the trait and its plasticity.

While population structure is often neglected in deterministic evolutionary models of plasticity^35^ (reviewed by Berringan and Scheiner^59^) or examined only in the settings of multi-locus or phenotypic evolution of plasticity^37–40^, it appears to be the key to generating the balancing effect of heterogeneous indirect selection and the consequent rapid evolution at a plasticity modifier locus. Such a population structure is likely to arise as organisms such as plants disperse passively over patchy habitats^41,42,60^, under ecological or evolutionary traps^61^, or due to anthropogenic disturbance^43,44^ such as global transport^45–47^, making the mechanisms applicable to natural scenarios where plasticity could rescue populations from extinction^62^ or aid in their invasive success^9^. Therefore, this study expands on an important, underexplored, and plausible biological scenario where the rapid molecular evolution of phenotypic plasticity could aid in rapid adaptation, and the findings further highlight the importance of population structure in understanding the evolution of phenotypic plasticity.

Cost to plasticity is also an important factor to rapid adaptation. Under bi-directional migration, the strength of the proposed mechanism depends on the cost to plasticity^5,15–17,63^. Costs to plasticity have been difficult to determine^64^ (also reviewed in Murren et al.^30^), although the fact that plasticity may involve complex structures and be evolutionary maladaptive, as evidenced by its disappearance following assimilation, points to nontrivial costs^5,15–17,28,29^. Another novel implication of this study is that the cost of plasticity is an important evolutionary factor, not only in whether plasticity can evolve in populations^15,30^ but also in whether selection leads to balanced polymorphism for plasticity across structured populations. That is, in the absence of the cost, plasticity would be at worst neutral in ancestral demes while advantageous in adverse environments. This situation would easily lead to its fixation in the metapopulation through immigration from adverse environments, creating a *reductio ad absurdum* in which plasticity is favored in all traits. Such a cost can therefore maintain genetic differentiation and polymorphism across a structured population and promote rapid adaption through plasticity. Our study, therefore, argues that negative selection due to the cost of plasticity might be an important condition for mechanisms of plasticity that can rapidly evolve when migration is bi-directional. However, cost is not required to maintain the capacity for rapid adaptation when there are unidirectional migration patterns or source-sink dynamics, because migration-selection balance can be maintained in recipient demes without modifiers from the ancestral demes. Therefore, while cost is not required for rapid adaptation, it nonetheless enables plasticity to rapidly evolve in a wider range of conditions.

Phenotypic plasticity is an important biological phenomenon that promotes population persistence in the face of environmental change. While many theoretical studies have greatly advanced our understanding of the evolution of plasticity^35–40,58^, the basis for the rapid plastic response is a qualitatively distinct phenomenon that requires further theoretical investigation. Given that the main role of plastic response is to protect populations from the negative effects of selection when conditions change, its ability to rapidly arise seems to be key to its benefit. However, so far, models recovering rapid plastic response assumed evolution across multiple loci or depend on high mutation rates^65^. These same mechanisms could just as well drive fast adaption at the directly selected structural loci, quickly rendering plasticity obsolete. Balanced polymorphism represents another venue for the rapid evolution of plastic or non-plastic response, via protected balanced polymorphisms at the plasticity modifier locus. Gulisija et al.^13,14^ offered a plausible scenario for balanced polymorphism and rapid molecular evolution of phenotypic plasticity that requires predictably changing temporally varying environments. However, by the nature of temporal change this scenario is limited predominantly to evolution in seasonally evolving organisms^66,67^ and makes specific assumptions about genetic architecture and recombination. As biodiversity faces increasing rates and magnitudes of environmental change^68^ due to global climate change, habitat conversion, invasive species transport, range shift, and many other natural and anthropogenic stressors, the rapid evolution of phenotypic plasticity needs to be better understood in order to predict future patterns of biodiversity. Mechanisms akin to the one uncovered in this study could greatly advance modeling and understanding of the effects of global climate change on persistence in perturbed habitats or forced dispersal to novel habitats.

Our study, however, introduces two caveats. The population genetics approach applied here allowed us to achieve an elegant and explicit examination of molecular evolution at a plasticity locus in a structured population in patchy habitats, unlike typically examined in optimality and quantitative genetics models^64^. However, in reality, plasticity may be controlled by more than two loci^64^, and the presence of many loci may result in interactions that affect evolution at a modifier-target pair. In addition, the evolution of plasticity depends on the trait’s constitutive loci, and might further be affected by the number of such non-plasticity loci as well^69^. Therefore, extending the proof-of-concept results reported here to multi-locus case remains open for future study.

Furthermore, the findings in this study apply to evolution in patchy habitats when recurrent mutation rates are low, and argue that rapid evolution of plasticity can occur as conditions change because of pervasive balanced polymorphism at plasticity modifier loci. Another caveat of our findings is the assumption that mutation rates are low, or by extension that constitutive adaptation is unlikely or not possible, and hence plasticity will aid in population persistence. This assumption excludes cases characterized by high beneficial mutation rates at loci under direct selection. In fact, balanced polymorphism can occur either at the plasticity locus or at the target locus, but not both. Indeed, following adaptation in all demes under our model, the polymorphism in phenotypic plasticity is removed from populations. Therefore, this model does not apply to the rapid evolution of phenotypic plasticity in populations adapted to local conditions. Such cases are subject to future studies of the evolution of plasticity needed to fully elucidate this important biological phenomenon.

### Supplementary Information

Supplementary Information 1.

Supplementary Information 2.

## Data Availability

The code used in this study is freely available on Github at https://github.com/nawsheentpromy/RapidEvolutionNTP.
