# Supplementary material for: Rapid evolution of phenotypic plasticity in patchy habitats
Source: Sci Rep. 2023 Nov 6;13:19158. doi: 10.1038/s41598-023-45912-8 (PMC10628295; doi:10.1038/s41598-023-45912-8)
Supplement: Supplementary file 1 — Supplementary Information 1. [file 41598_2023_45912_MOESM1_ESM.pdf]

Supplementary Information 2 – Deterministic Model and Stability Analysis

Rapid evolution of phenotypic plasticity in patchy habitats

Nawsheen T. Promy[1],

Mitchell Newberry[2,3], and

Davorka Gulisija[1,3*]

[1] Department of Computer Science, University of New Mexico, USA

[2] Center for the Study of Complex Systems, University of Michigan, USA

[3] Department of Biology, University of New Mexico, USA

[*] Corresponding author: dgulisija@unm.edu

Davorka Gulisija

219 Yale Boulevard NE

3566 Castetter Hall

Albuquerque, NM 87131

United States

Continuous Deterministic Model and Local Stability Analysis

To investigate the stability of polymorphic equilibria we observe in finite populations simulations, we model the dynamics in a continuous-time infinite population limit with the same parameters and fitness effects as described in Model and Methods Eqs. (1)-(5) for the case of symmetric selection and plasticity effect. We are primarily interested in the evolution of the plasticity locus before adaptation in the novel maladapted deme occurs, i.e. when non-plastic, constitutive adaptation is slow. Therefore, we simplify our analysis with the reasonable assumption that the plasticity allele *M* circulates within a meta-population that is monomorphic for the ancestral (*a*) allele at the target locus. This allows further simplification, as recombination has no effect on haplotype frequencies, i.e. the linkage disequilibrium coefficient $D = 0$. We may thus track the full of state of both demes as the frequency $x_{M1,t}$ of the plasticity modifier allele *M* in deme 1 (the ancestral deme in which the *a* allele is adaptive) and $x_{M2,t}$ in deme 2 (the deme in the adverse environment in which *a* is maladaptive). In the absence of temporal environmental variation and under constant, symmetric selective pressures of opposite direction, with $s_1 = s$ and $s_2 = -s$, where $s > 0$, the time evolution of the frequencies $x_{M1,t}$ and $x_{M2,t}$ can then be fully described by an autonomous dynamical system on the $x_{M1,t}$, $x_{M2,t}$ (henceforth x_1, x_2) state space [1].

We specify continuous-time dynamics by writing formulas for $\dot{x}_i = dx_i/dt$. The difference in growth rate dx_i/dt due to selection is proportional to selection coefficient *s*, the selection-buffering effect of phenotypic plasticity, *p*, and the frequencies of the alleles *M* and *m*. Then the selective term of dx_i/dt is $-spx_1(1 - x_1)$ in the ancestral deme and $spx_2(1 - x_2)$ in the derived deme. This is because in order for the *M* allele to increase in deme 1, first an *m*-carrying individual must be chosen to die, with probability $(1 - x_1)$, then an individual of *M* allele must be chosen to reproduce with probability x_1. Since selection is differential intrinsic growth per generation, this

process of gaining M individuals occurs at a rate of $1 + s(1 - p)$, whereas the reverse process (M replaced by m) occurs at a rate $1 + s$. Hence the net rate of increase in M is

$1 + s(1-p) - (1+s) = -sp$ in the ancestral deme (1, adapted), and

$1 - s(1-p) - (1-s) = sp$ in the derived deme (2, maladapted).

Likewise, the exchange of individuals by migration happens at a rate of e/IN per individual per generation, where $e = e_1 = e_2$ and $N = N_1 = N_2$ are the number of migrants to and the total population of a deme. Deme 1 gains M individuals if an individual of type m is replaced by an individual of type M from deme 2. This happens with a probability equal to the frequency of m in deme 1 times the frequency of M individuals in deme 2. The full process of gaining M individuals thus happens at a rate $(e/IN)\,(1 - x_1)\,x_2$ in deme 1 and $(e/IN)\,(1 - x_2)\,x_1$ in deme 2. The process of losing M individuals by the reverse process happens at the rate $(e/IN)\,x_1\,(1 - x_2)$ in deme 1 and analogously for deme 2. Combining these rates of gain and loss of M individuals, we can write the continuous-time dynamics as:

$$\dot{x}_1 = -spx_1(1 - x_1) + \frac{e}{IN}(x_2 - x_1) \qquad (10)$$

$$\dot{x}_2 = spx_2(1 - x_2) + \frac{e}{IN}(x_1 - x_2) \qquad (11)$$

The result is a two-dimensional nonlinear dynamical system. The expressions make it clear that there are effectively only two parameters to the symmetric continuous-time model, sp and e/IN, each with "rate" dimensions, 1/time. We denote these effective parameters α and β, where $\alpha = sp$ can be interpreted as controlling the strength of selection on the plastic type relative to the non-plastic type in each habitat and $\beta = e/IN$ as controlling the rate of migration as a nondimensional fraction of the total population per unit time. These are both positive since selection is stipulated to favor a in deme 1 and disfavor a in deme 2, p is between 0 and 1, and the rate of migration events cannot be negative.

We solve for equilibrium states (x_1^*, x_2^*) by setting the time derivatives $\dot{x}_1$ and $\dot{x}_2$ in the Eqs. 10-11 equal to 0. We first note some special cases in terms of the parameters. If the migration parameter β = 0, then the equations become two decoupled one-dimensional dynamics representing the independent dynamics in each deme. In this case, there are equilibria when x_1^* and x_2^* take values either 0 or 1. The equilibrium in x is stable if the $d\dot{x}/dx$ < 0. This is true if x_1= 0 and if x_2= 1. These equilibria can be summarized with the statement that without mutation, the *M* allele will eventually be lost in deme 1 unless it is already fixed and eventually fix in deme 2 unless it is never present.

Likewise, if the selection parameter α is 0, there is either no selection, s = 0, or no effect of plasticity p = 0. In this case, there is a line of interior equilibria corresponding to $x_1 = x_2$. This line is neutrally stable, as we can see taking the change of variables $u = x_2$ - x_1, $v = x_1$+ x_2. This transformation uncouples the dynamics, which become:

$$\dot{u} = \dot{x}_2 - \dot{x}_1 = \frac{e}{IN}(-u - u) = -\frac{2e}{IN}u$$
$$\dot{v} = \dot{x}_1 + \dot{x}_2 = \frac{e}{IN}(x_2 - x_1 + x_1 - x_2) = 0$$

We see that the derivative in u is negative, whereas $\dot{v} = 0$, so that its derivative is zero at all orders. The frequencies, therefore, approach the line x_1 = x_2 on the perpendicular trajectories x_1 + x_2 = *const*.. In real terms, if there is no selection or no buffering effect, the *M* allele has no effect, and the total amount of *M* allele in both demes stays constant while migration tends to homogenize the fraction of *M* in both demes, equalizing the two frequencies. In the corresponding stochastic scenario, migration thus tends to homogenize the allele frequencies across demes while drift neutrally varies the total fraction of *M* in the metapopulation until the eventual extinction or fixation of *M* with fixation probability equal to its initial total frequency $(N_1x_1 + N_2x_2)/(N_1 + N_2)$.

A variation on the case of no selection occurs if α is not zero but $\alpha \ll \beta$. This is case in which selection is effectively negligible relative to migration, but it has can have qualitatively different equilibria depending on the nature of costs and benefits to plasticity. In this case, $\dot{v} = \alpha(x_2(1-x_2) - x_1(1-x_1))$, which is not generally zero. However, we can regard the dynamics in $\dot{u}$ as much faster than $\dot{v}$, and so we may assume that trajectories lie close to the line $x_1 = x_2 = x$, where x is the homogenized frequency in both populations. In this case, again $\dot{v} = \alpha\big(x(1-x) - x(1-x)\big) = 0$ and $x_1 = x_2$ forms a line of neutrally-stable equilibria that the system rapidly approaches. That is, if mutation is so strong that selection on a given genotype from generation to generation become effectively random, the result is identical to neutrality. However, this depends sensitively on the symmetric assumptions around s and p. Neutrally stable equilibria are often a clue to look for sensitive dependence on symmetries or specific parameter values, which is called structural instability in dynamical systems theory [1]. In our case, neutral stability depends on the symmetry in selection and plasticity s and p across habitats. If instead, we allow the effects of selection and plasticity s_i and p_i to vary between habitats, $\dot{v}$ becomes $s_2p_2x_2(1-x_2) - s_1p_1x_1(1-x_1))$. In this case even when $x_1 = x_2 = x$, then $\dot{v}$ evaluates to the nonzero $2(s_2p_2 - s_1p_1)x(1-x)$. The only equilibria are then $x_1^* = x_2^* = 1$ and 0, which are either stable and unstable or unstable and stable respectively depending on the sign of the coefficient $(s_2p_2 - s_1p_1)$. This coefficient represents the net cost or benefit to plasticity averaged over both environments, which is unlikely to be zero in general. If this net selection coefficient is non-zero, the plasticity allele will either fix or be lost in both populations depending on whether the benefits outweigh the costs on average. Because this result assumes effective homogenization of the populations through migration, it illustrates how the protection of balanced polymorphism requires limited migration.

With α, $\beta > 0$, corresponding to the presence of some selection and migration, there is only one interior equilibrium within the state space x_1,x_2 $\in$ [0, 1]. The only boundary equilibria are at $x_1 = x_2 = 0$ or 1, corresponding to fixation or loss of the *M* allele from both demes. In this case all terms of both Eqs. 10-11 go to zero. It is easy to prove that this is the only boundary equilibrium: the first terms of Eqs. 10-11 are always zero on the boundary, and so setting the second terms to zero yields $x_1 = x_2$.

Linear stability analysis gives the stability of the boundary equilibria, corresponding to fixation or loss from the population. The local linearization around the fixed point $\dot{u}$, $\dot{v}$ in terms of the variables $u = x_1 - x_1^*$, $v = x_2 - x_2^*$ is given by the Jacobian J evaluated at the fixed point x_1^*, x_2^*,

$$\begin{pmatrix} \dot{u} \\ \dot{v} \end{pmatrix} = \begin{pmatrix} -\alpha(1 - 2x_1^*) - \beta & \beta \\ \beta & \alpha(1 - 2x_2^*) - \beta \end{pmatrix} \begin{pmatrix} x_1 - x_1^* \\ x_2 - x_2^* \end{pmatrix} . \qquad (12)$$

The determinant of the Jacobian is the product of the eigenvalues. At $x_1^* = x_2^* = 0$, the determiant is det(J) = $(-\alpha - \beta)$ $(\alpha - \beta) - \beta^2 = -\alpha^2$, which is always negative. The determinant at $x_1^* = x_2^* = 1$ is also $-\alpha^2$, since the factors $(1 - 2x_1^*)$ are simply ± 1 depending on whether we consider the equilibrium at $x_1^* = 0$ or 1 and thus have no effect on the determinant.

The negative determinant of the linearization unequivocally indicates that both fixation and loss correspond to an unstable equilibrium. In this case the equilibria are saddle-nodes, meaning both eigenvalues are non-zero and have opposite sign. This means the system evolves towards equilibrium in one direction and away from equilibrium in another. Indeed, the eigenvalues $\lambda_{1,2}$ are given by the solution to the characteristic polynomial $\lambda^2 - \mathrm{trace}(J)\lambda + \det(J) = 0$. The trace of J is -2β at either equilibrium, and so the eigenvalues are

$$\lambda_{+,-} = -\beta \pm \sqrt{\beta^2 + \alpha^2}$$

at each equilibrium. The formula makes clear that the eigenvalues have opposite sign, and that fast eigendirection is towards the equilibrium. Although the eigenvalues are the same at each equilibrium, the corresponding eigenvectors for the unstable manifold of the saddle differ, and are equal to

$$\begin{pmatrix} \beta \\ \sqrt{\beta^2+\alpha^2} \pm \alpha \end{pmatrix}$$

taking the positive sign of α at $x_1^* = x_2^* = 0$ and the negative at $x_1^* = x_2^* = 1$. At either equilibrium, both components of the eigenvector have the same sign, and so the eigenvector, and hence the direction of evolution of the system, points into the interior of the state space, steeper at the (0, 0) equilibrium and shallow at (1, 1).

In physical terms, this means that the *M* allele can invade a population otherwise monomorphic for *m*, and likewise the *m* allele can invade a monomorphic *M* population, a traditional criterion for stable coexistence in ecology. If the *M* allele is invading, its frequency rises more quickly in deme 2, with the ratio of $\dot{x}_2/\dot{x}_1 = \alpha/\beta + \sqrt{1+\alpha^2/\beta^2} > 1$ that depends on the relative strength of selection versus migration. Conversely if the *m* allele is invading, its frequency rises more quickly in deme 1, with the ratio of rates $\dot{x}_2/\dot{x}_1 = \sqrt{1+\alpha^2/\beta^2} - \alpha/\beta$, which is between 0 and 1 by the Pythagorean theorem. As migration becomes much larger than selection, these relative rates converge to 1, the eigenvectors converge to (1, 1), a stable manifold approaches the line $x_1 = x_2$, and movement along the stable manifold goes to zero. In this limit, the unstable eigendirection of the saddle-node converges onto the line $x_1 = x_2$ and progress along the manifold slows to a halt, with a bifurcation to a line of neutrally stable equilibria when $\alpha = 0$.

If the only equilibria on the system boundary are unstable, this suggests a stable interior equilibrium. This equilibrium is given by setting Eqs. 10 and 11 to zero. The result is a polynomial with the only one positive real root:

$$x_1^* = \frac{\alpha+2\beta-\sqrt{\alpha^2+4\beta^2}}{2\alpha},\ x_2^* = x_1^*\left(\frac{\alpha+\sqrt{\alpha^2+4\beta^2}}{2\beta}\right) \quad (13)$$

The equilibrium frequencies in the two demes depend only on the ratio of α and β, with $\alpha = sp$ and $\beta = e/IN$. This can be seen by taking $c = \sqrt{\alpha^2 + 4\beta^2}$ and then $a = \alpha/c$, $b = 2\beta/c$. Then a, b, and c are the legs and hypotenuse of a right triangle (by the Pythagorean theorem) and

$$x_1^* = \frac{1}{2}\left(1 + \frac{\cos(\theta) - 1}{\sin(\theta)}\right), \qquad x_2^* = x_1^* \frac{\sin(\theta) + 1}{\cos(\theta)}, \qquad \theta = \tan^{-1}(\alpha/2\beta)$$

We can also discern from these expressions that the equilibrium x_1^* is bounded between 0 and 1/2 while x_2^* is bounded between 1/2 and 1. The equilibrium at $x_1^* = x_2^* = 1/2$ is attained only in the limit that β goes to infinity with $\alpha > 0$. The interior equilibrium is always stable, since for $x_1^* < 1/2$ and $x_2^* > 1/2$, the determinant of the Jacobian (Eq. 12) is always positive while the trace is always negative, indicating two negative eigenvalues and therefore an exponential return to equilibrium after perturbation in any direction. The eigenvalues are nonzero provided α and β are nonzero and increase with the magnitudes of α and β providing their ratio is held constant. As migration becomes much larger than selection with $\alpha > 0$ however, the eigenvalues and therefore rate of return to equilibrium go to zero as the equilibria approach frequency 1/2, increasingly resembling the line of neutrally-stable equilibria when $\alpha = 0$. This equilibrium is also structurally unstable, however, and when we relax the assumption of symmetry between the two populations, any small imbalance in costs and benefits to plasticity averaged across the two populations drives equilibria towards fixation or loss of the plasticity allele. This implies that limited migration is required to protect balanced polymorphism, and with less migration, larger discrepancies in selection and

plasticity between populations or average costs and benefits to plasticity can still support balanced polymorphism.

In summary, for non-zero, symmetric rate parameters sp and e/IN, a stable polymorphism exists in both populations, and both the m and M alleles are able to invade from low frequencies. The equilibrium frequencies depend only on the parameter ratio $spIN/e$, and the equilibrium exists whenever this ratio is defined and greater than zero. The rate of approach to equilibrium depends on the magnitudes of sp and e/IN, as well as their ratio, and so the waiting time for the M allele to fix or be lost depends on the strength of drift as well as the parameter values. Fixation or loss will happen most readily if e/IN, is large and $spIN/e$ is small, since this brings the system closest to the neutrally stable equilibria $x_1^* = x_2^*$ where the alleles experience selection in both populations and quasi-neutrality converges to true neutrality.

References:

[1] Strogatz, S. H. *Nonlinear dynamics and chaos with applications to physics, biology, chemistry, and engineering.* (Westview Press, 2nd edition, 2015).
