# Supplementary material for: Rapid evolution of phenotypic plasticity in patchy habitats
Source: Sci Rep. 2023 Nov 6;13:19158. doi: 10.1038/s41598-023-45912-8 (PMC10628295; doi:10.1038/s41598-023-45912-8)
Supplement: Supplementary file 2 — Supplementary Information 2. [file 41598_2023_45912_MOESM2_ESM.pdf]

Supplementary Information 2 – Supplementary Figures

Rapid evolution of phenotypic plasticity in patchy habitats

Nawsheen T. Promy[1],

Mitchell Newberry[2,3], and

Davorka Gulisija[1,3*]

[1] Department of Computer Science, University of New Mexico, USA

[2] Center for the Study of Complex Systems, University of Michigan, USA

[3] Department of Biology, University of New Mexico, USA

[*] Corresponding author: dgulisija@unm.edu

Davorka Gulisija

219 Yale Boulevard NE

3566 Castetter Hall

Albuquerque, NM 87131

United States

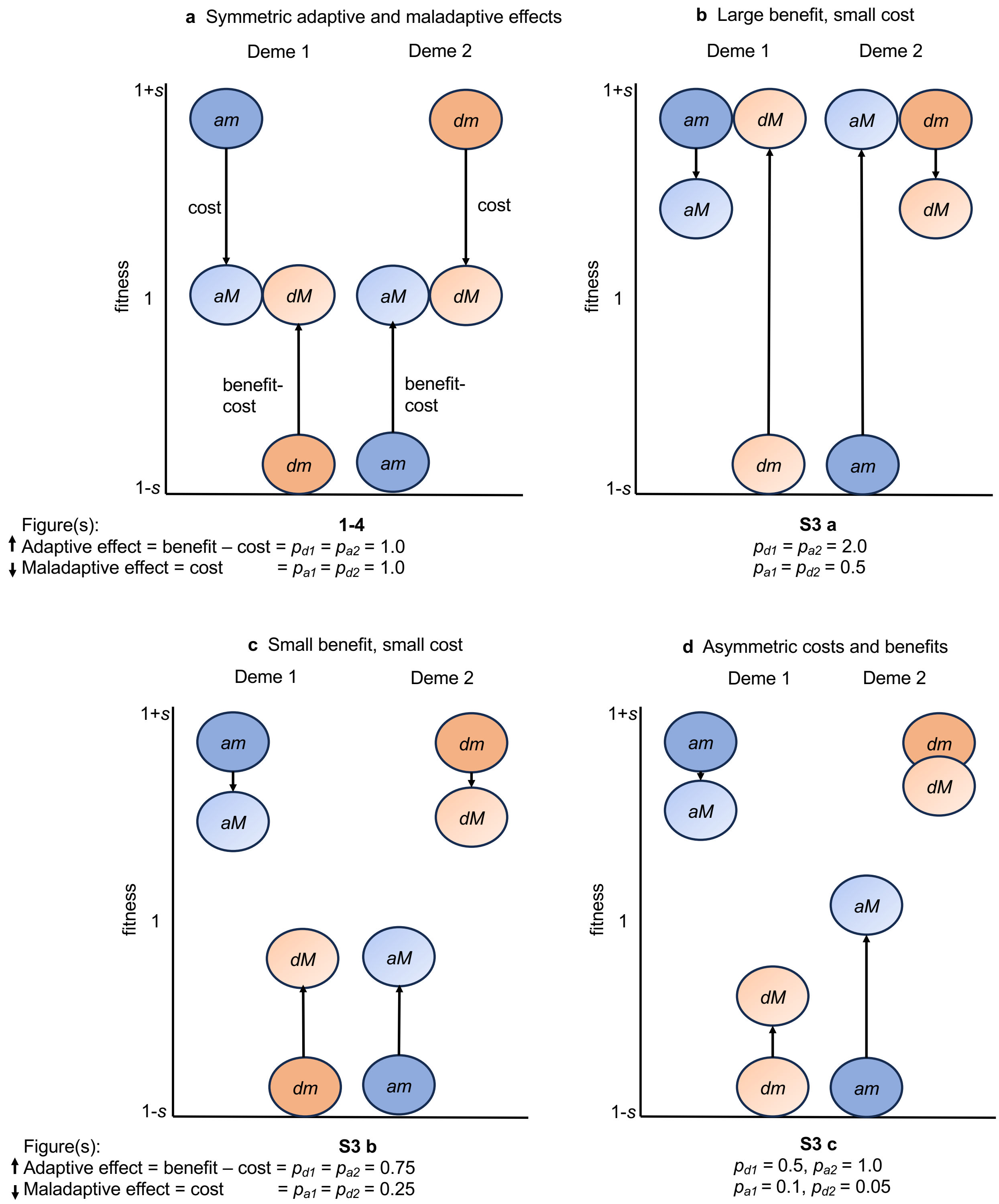

Figure S1. Illustration of the relative fitness effects of various benefits and costs of plasticity under the model. (**a**) illustrates the symmetric fitness scheme used to produce figures 1-4 in the main text, while (**b**), (**c**), and (**d**) illustrate asymmetric adaptive and maladaptive effects of plasticity, assuming small cost, used to generate Figure S3 a, b, and c.

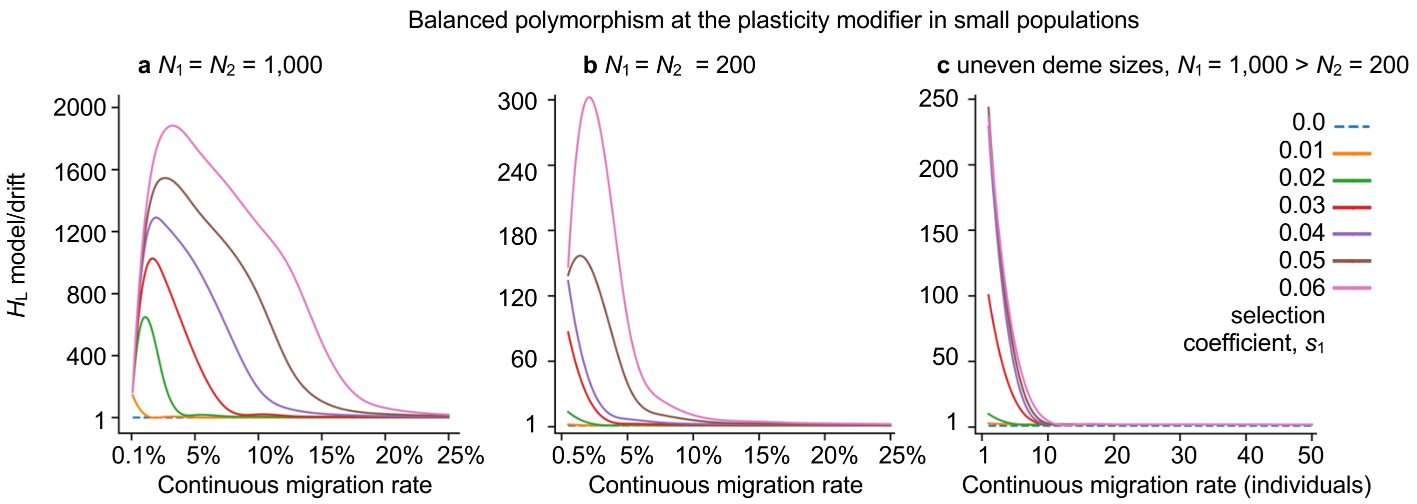

Figure S2. **Balanced polymorphism at the plasticity modifier locus in small populations.** The levels of heterozygosity at the plasticity modifier locus (>1 = exceeds neutrality, broken line) in structured populations when (**a**) $N_1 = N_2 = 1000$ (**b**) $N_1 = N_2 = 200$, and (**c**) uneven population sizes, $N_1 = 1000 > N_2 = 200$, with varying symmetric opposing selective pressures ($s = s_1 = -s_2 = 0.0, 0.01, 0.02, 0.03, 0.04, 0.05, 0.06$). Forward-in-time computer simulations were conducted over 100N generations and 1000N replicate runs per parameter combination using plasticity effect $p = 1.0$ and recombination rate $r = 0.5$. The curves were smoothed using a spline function and perfectly align with the data points.

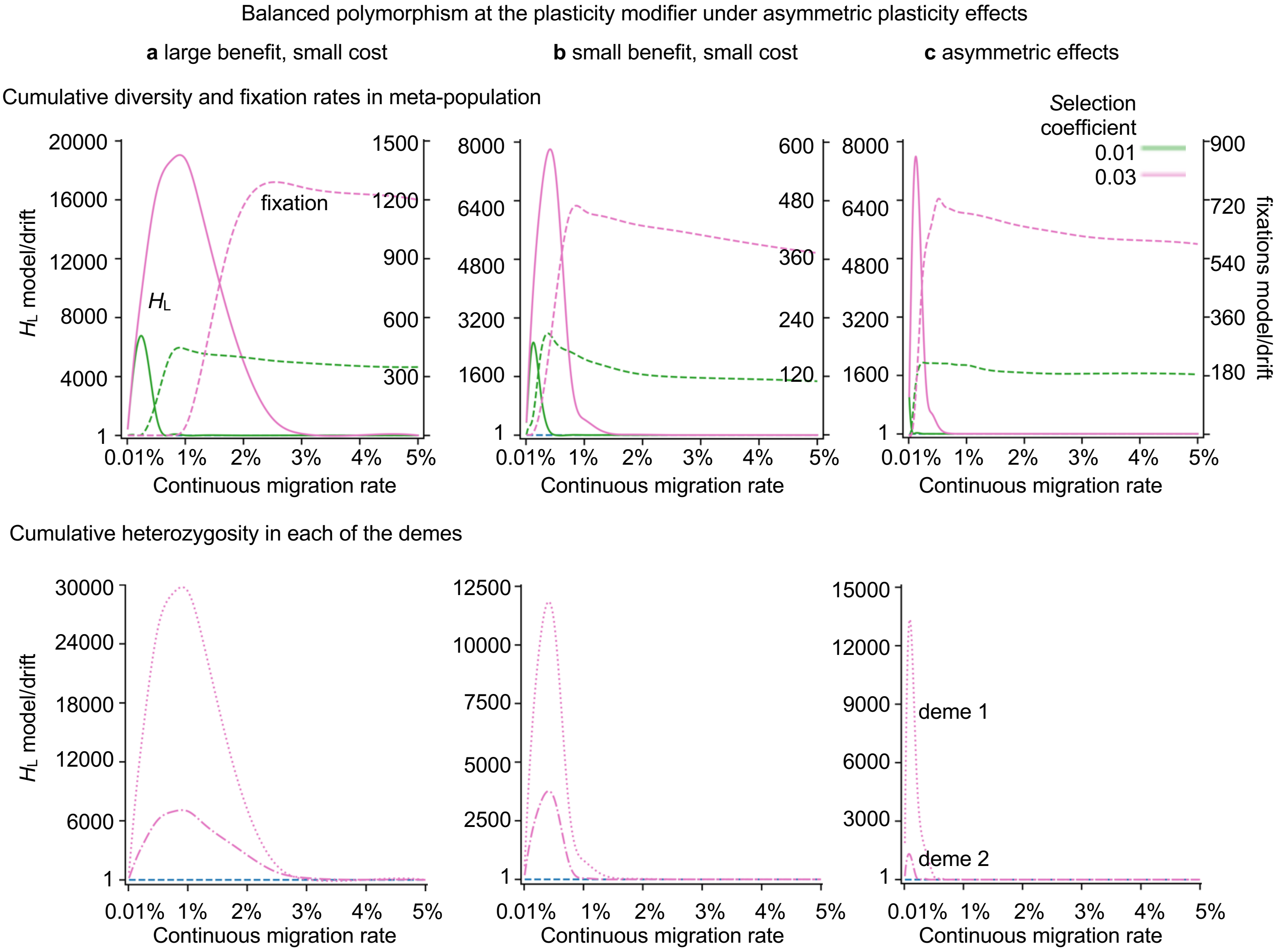

Figure S3. **Balanced polymorphism at the plasticity modifier locus with asymmetric plasticity effects and small cost.** The levels of heterozygosity at the plasticity modifier locus in a metapopulation (top row) and in each of the demes (bottom row), with fixation rate of the plasticity modifier allele relative to neutrality (top row) (>1 = exceeds neutrality) in structured populations when (**a**) there is large benefit to plasticity ($p_{d1} = p_{a2} = 2.0$) and a small cost ($p_{a1} = p_{d2} = 0.5$), as illustrated in Figure S1 **b**, (**b**) there is a small benefit ($p_{d1} = p_{a2} = 0.75$) and small cost ($p_{a1} = p_{d2} = 0.25$), as illustrated in Figure S1 **c**, and (**c**) with variable benefits ($p_{d1} = 0.5$ and $p_{a2} = 1.0$) and variable minimal costs ($p_{a1} = 0.1$ and $p_{d2} = 0.05$), as illustrated in Figure S1 **d**, with varying symmetric opposing selective pressures ($s = s_1 = -s_2 = 0.0$, 0.01, or, 0.03). Forward-in-time computer simulations were conducted over 2 million generations and 20 million replicate runs per parameter combination where recombination rate $r = 0.5$ and $N_1 = N_2 = 10000$. The curves were smoothed using a spline function and align with the data points.

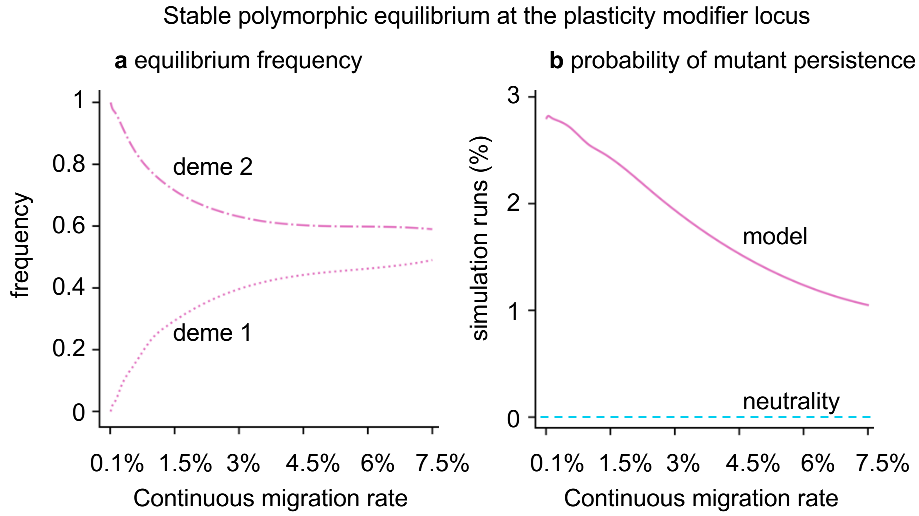

Figure S4. **Properties of the stable intermediate equilibria.** Stable equilibrium frequencies of the plasticity allele (a) and the fraction of simulation runs in which the mutant modifier allele persists for the simulation duration following single mutant introduction (b). Forward-in-time computer simulations were conducted across a range of varying migration rates using 4000000 replicate runs per parameter combination, assuming symmetric opposing selective pressures, $s = s_1 = -s_2 = 0.03$, symmetric plasticity effect $p = 1.0$, and recombination rate, $r = 0.5$. Stable polymorphic equilibria were observed at 10000 ($N/2$) generations and confirmed at $2N$ and $4N$ generations.

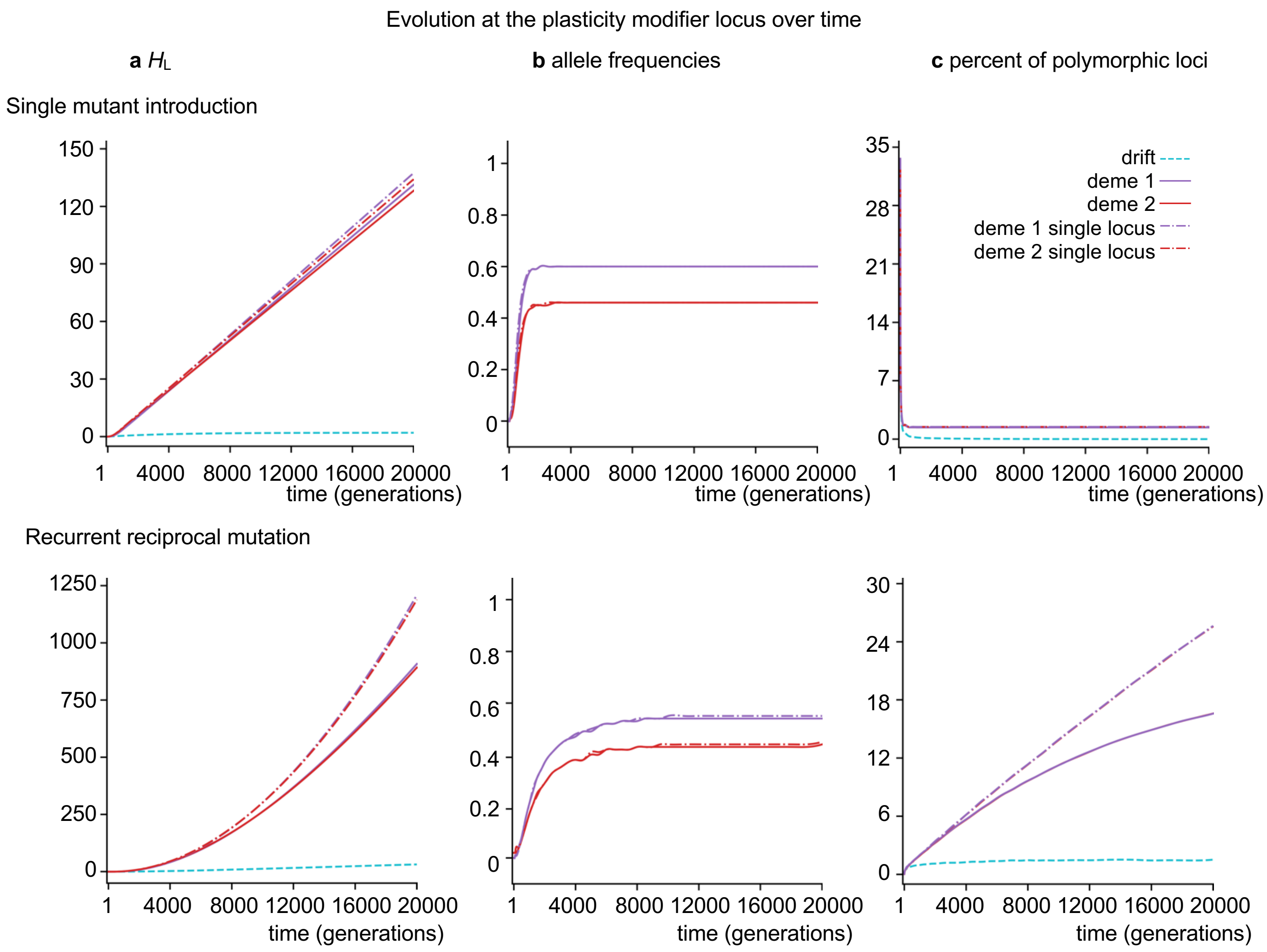

Figure S5. **Characteristics of the polymorphic equilibrium at the plasticity modifier locus in a structured population over time.** Columns illustrate (**a**) the continuous increase in diversity (H_L) due to the attainment of equilibrium, (**b**) stable allele frequency among polymorphic loci, and (**c**) runs that reach the equilibrium remaining polymorphic over long periods (given as a percent of runs containing segregating loci). These characteristics are shown for each deme under a single (dashed lines) and two-locus (full lines) dynamics, and under a single mutant introduction (top row) and recurrent mutation (bottom row). The single locus dynamics were simulated by assuming a continuously monomorphic target locus, disallowing mutation at the target locus. Recurrent mutation changed a random allele copy in one or the other population into the other allele with a chance of 0.1% per generation per locus ($N\mu = 0.001$ and $\mu = 2.5^{x}10^{-7}$). Forward-in-time computer simulations were conducted over 20000 generations and 8000000 replicate runs (single mutant introduction) or 100000 (recurrent mutation) per model and assumed symmetric opposing selective pressures $s = s_1 = -s_2 = 0.03$, reciprocal migration of 100 individuals between demes, recombination rate $r = 0.5$, $p = 1.0$, and $N_1 = N_2 = 2000$. The curves were smoothed using a spline function and align with the data points.
